# *Sanicula fengjiensis* (Apiaceae), a New Species From China Revealed by Integrative Morphological and Molecular Evidence

**DOI:** 10.1002/ece3.74390

**Published:** 2026-09-29

**Authors:** Hui‐Min Li, Ji‐Min Liao, Xu‐Dong Ma, Chun‐Feng Song

**Affiliations:** ^1^ Jiangsu Provincial Key Laboratory for Plant Taxonomy, Resource Conservation and Utilization, Institute of Botany Jiangsu Province and Chinese Academy of Sciences (Nanjing Botanical Garden Memorial Sun Yat‐Sen) Nanjing Jiangsu China

**Keywords:** China, new species, phylogeny, plastome, *Sanicula*

## Abstract

*Sanicula fengjiensis* (Apiaceae), a new species discovered in Fengjie County, Chongqing Municipality, southwestern China, is described and illustrated. It is morphologically most similar to *S. caerulescens* in having pseudo‐racemose inflorescences, but differs in having predominantly white petals (vs. blue to purple) and distinctive fruit ornamentation, with prickles absent, the ribs basally fused into a thin layer, and the valleculae occasionally bearing sparse scales or tubercles (vs. fruits sparsely to densely covered with short, straight spinous bristles). Quantitative morphological analyses further distinguished *S. fengjiensis* from *S. caerulescens* and the phylogenetically proximate *S. lamelligera* and *S. orthacantha*. In particular, the maximum height of fruit‐surface appendages in *S. fengjiensis* (0.033–0.101 mm) showed no overlap with the observed ranges of the other three species. The complete plastomes of four sampled accessions of *S. fengjiensis* were all 155,501 bp in length and exhibited the typical quadripartite structure. Plastome and nrDNA phylogenies based on complete plastome sequences and combined ITS and ETS sequences, respectively, consistently recovered the sampled accessions of *S. fengjiensis* as a clade, although its relationships with other *Sanicula* species differed between the two datasets. In addition to the extant population in Fengjie, a historical herbarium specimen collected from Yichang, Hubei Province, in 1963 is attributable to the new species. The combined morphological and phylogenetic evidence supports the recognition of *S. fengjiensis* as a distinct species.

## Graphical abstract

*Sanicula fengjiensis* is described as a new species from Chongqing, China, based on integrative morphological and molecular evidence. Quantitative morphological analyses, together with distinctive inflorescence and fruit characters, clearly differentiate it from the morphologically similar *S. caerulescens* and the phylogenetically proximate *S. lamelligera* and *S. orthacantha* . Plastome and nrDNA phylogenies consistently recover *S. fengjiensis* as a distinct lineage, further supporting its recognition as a separate species.

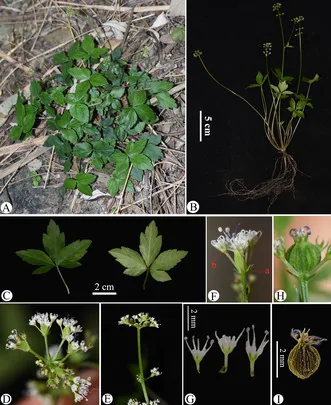

## 1 Introduction

*Sanicula* L. (Apiaceae, subfamily Saniculoideae) comprises ca. 42 taxa and is widely distributed from East Asia to North America (Van Wyk et al. 2013; Li et al. 2023). The genus has long been regarded as taxonomically complex due to its considerable morphological variation in rhizomes, leaves, inflorescences, and fruits, and it has been considered a relatively primitive lineage within subfamily Saniculoideae (Shan and Constance 1951; Hiroe 1979; Calviño et al. 2008; Wen et al. 2020; Xie et al. 2022). Over the past two decades, only four species and one variety have been newly described in the genus (Kamelin et al. 2002; Xie et al. 2019; Kenny et al. 2022; Song et al. 2024). Meanwhile, seven species and one variety have been reduced to synonymy (Li et al. 2022, 2024; Li and Song 2022; Li, Zhou, et al. 2025; Li, Zhu, et al. 2025). China is one of the major centers of distribution for *Sanicula*, harboring ca. 16 species (Li et al. 2022, 2024; Li and Song 2022; Song et al. 2024; Li, Zhou, et al. 2025; Li, Zhu, et al. 2025).

In taxonomically complex plant groups, taxa may remain overlooked or misidentified when morphological characters provide insufficient resolution (Duminil and Di Michele 2009; Bebber et al. 2010; Goodwin et al. 2015), particularly when available specimens lack the reproductive structures required for reliable identification (Sullivan and Nazaire 2022). Fruit morphology provides important diagnostic characters for species identification and delimitation in *Sanicula* (Shan and Constance 1951; Pryer and Phillippe 1989; Chen et al. 2019; Li et al. 2023). Recent studies have increasingly incorporated both plastome and nuclear ribosomal DNA (nrDNA) data to provide complementary phylogenetic evidence for species delimitation (Shen et al. 2025; Zhang et al. 2025). Therefore, integrating detailed morphological examination of numerous specimens, particularly of reproductive characters, with molecular phylogenetic evidence can help distinguish overlooked taxa from intraspecific variants and identify previously misidentified specimens.

During our botanical expeditions to Fengjie County, Chongqing Municipality, southwestern China, from 2022 to 2024, we encountered an unusual population of *Sanicula* that exhibited morphological features resembling *S. caerulescens* Franch., a species widely distributed in various regions of China and Vietnam (Li et al. 2024). These plants shared pseudo‐racemose inflorescences with *S. caerulescens* , a key characteristic of the species. However, upon further examination, we discovered that these plants differed significantly in fruit morphology, a crucial diagnostic feature in *Sanicula*. These morphological differences led us to hypothesize that the Fengjie population represented a previously overlooked taxon rather than an intraspecific variant of *S. caerulescens* . Subsequent plastome and nrDNA phylogenetic analyses further supported the distinctiveness of the Fengjie population, while the nrDNA phylogeny indicated particularly close relationships with *S. lamelligera* Hance and *S. orthacantha* S. Moore. These results prompted us to extend the morphological comparison beyond *S. caerulescens* to include these phylogenetically proximate species.

During our examination of herbarium material, we also identified a historical specimen collected from Yichang, Hubei Province, in 1963 that was morphologically consistent with the Fengjie plants, particularly in its diagnostic fruit characters (Figure 2B). However, no extant plants have yet been relocated at the historical Yichang locality. We therefore treat this specimen as a historical herbarium record rather than evidence of a confirmed extant population. Here, we describe this previously overlooked taxon as *Sanicula fengjiensis* based on morphological evidence and plastome and nrDNA (ITS and ETS) phylogenetic analyses. We further compare it with the morphologically similar *S. caerulescens* and the phylogenetically proximate *S. lamelligera* and *S. orthacantha*, and clarify its phylogenetic placement and currently documented distribution.

## 2 Materials and Methods

### 2.1 Morphological Comparison

For morphological comparison, we examined physical specimens or high‐resolution images of specimens of *Sanicula caerulescens* from 64 herbaria, including CDBI, NAS, PE, and WUK (Li et al. 2024). We also observed living plants from five representative populations of *S. caerulescens* (Li et al. 2024), four representative populations of *S. orthacantha* (Li et al. 2022), three representative populations of *S. lamelligera* (Li and Song 2022) and the type population of *S. fengjiensis* in Fengjie county, Chongqing Municipality. The type population of *S. fengjiensis* was repeatedly observed over two consecutive years to assess the stability of its diagnostic morphological characters.

We also examined a historical herbarium specimen collected in 1963 from the vicinity of Huanglingmiao in Yichang, Hubei Province. To evaluate the current occurrence of *S. fengjiensis* in this area, we conducted targeted field surveys in Yichang in 2021, 2023, and 2024, including searches around Huanglingmiao and nearby habitats considered potentially suitable on the basis of the locality information recorded on the specimen label.

Living individuals of *S. fengjiensis* collected from the type population were transplanted to and cultivated at the Institute of Botany, Jiangsu Province and Chinese Academy of Sciences for repeated morphological observations under cultivation. The morphological description of *S. fengjiensis* was based on observations of herbarium specimens and living plants in the wild and in cultivation.

Morphological comparisons focused on inflorescence morphology, involucral‐bract morphology, calyx teeth, and fruit morphology. Ten quantitative characters were included in the statistical analyses: inflorescence length, number of primary branches, terminal capitate umbel diameter, involucral bract length and width, calyx tooth length and width, fruit body length and width, and maximum height of fruit‐surface appendages. Fruit‐surface ornamentation was additionally recorded as a qualitative character (e.g., prickly, scaly, tuberculate, or nearly smooth) but was not included in the PCA. Quantitative characters were measured from fully developed structures. Where multiple homologous structures were available from an individual, repeated measurements were averaged and the individual mean was used as the statistical unit. The number of inflorescences examined per individual, individual‐level measurements, and summary statistics for each taxon are provided in Tables S1 and S2.

Principal component analysis (PCA) was performed using individual‐level mean values for the ten quantitative morphological characters. Pairwise correlations among characters were first assessed using Spearman's rank correlation coefficients to evaluate potential redundancy among variables. Prior to PCA, all variables were centered and scaled to unit variance to account for differences in measurement units and numerical ranges. Because no extensive collinearity was detected and the correlated characters represented biologically distinct morphological features, all ten quantitative characters were retained in the PCA. PCA was conducted using the prcomp function in R v.4.5.3. Qualitative characters, including fruit‐surface ornamentation, were excluded from the PCA and evaluated separately in the morphological comparison (Table 2).

### 2.2 Phylogenetic Reconstruction

#### 2.2.1 Plant Materials, DNA Extraction, Plastome Sequencing, and NRDNA Amplification

Four individuals of *Sanicula fengjiensis* were sampled from the type population in Fengjie County for molecular analyses, including plastome sequencing and nuclear ribosomal ITS and ETS sequencing. Fresh and healthy leaf tissues were collected in the field and dried in silica gel. Voucher specimens were deposited in the Herbarium of the Institute of Botany, Jiangsu Province and Chinese Academy of Sciences (NAS), and the voucher numbers are listed in Table 1.

**TABLE 1:** Origins and voucher information for newly sequenced samples of *Sanicula* (four plastomes, 21 ITS, 20 ETS) and *Eryngium* (one ITS, one ETS), together with 15 publicly available plastome sequences of *Sanicula* and *Eryngium* retrieved from NCBI.

Order	Species	Localities	Coordinates	Altitude (m)	Voucher specimen	Cp genome	ITS	ETS
1	*E. alpinum*					NC_084330.1		
2	*E. foetidum*	Kunming, Yunnan			LHM1175		PZ195083	PZ228507
3	*E. foetidum*					NC_068234.1		
4	*E. planum*					MT561039.1		
5	*S. chinensis*					NC_085169.1		
6	*S. giraldii*					OR865883.1		
7	*S. hacquetioides*					NC_072641.1		
8	*S. odorata*					NC_061689.1		
9	*S. rubriflora*					OQ612642.1		
10	*S. rugulosa*	Fendou Village, Huize, Qujing, Yunnan	25.94214940° N/103.51156100° E	2292	LHM3002		PZ195092	PZ228515
11	*S. rugulosa*					NC_061561.1		
12	*S. brevispina*	Xixiangchi, Emeishan, Sichuan	29.5546723° N/103.3453723° E	2215–2400	LHM1054	NC_072645.1	PZ195084	PZ228508
13	*S. caerulescens*	Suijiang, Zhaotong, Yunnan	28.40185491° N/104.24369980° E	1307	SCF097		PZ195090	PZ228511
14	*S. caerulescens*	Yongshan, Zhaotong, Yunnan	28.2415796° N/103.63501° E	1538	SCF103		PZ195091	PZ228510
15	*S. caerulescens*	Jinyunshan, Beibei, Chongqing	29.8420075° N/106.3986754° E	850	LHM1005	NC_072644.1	PZ195089	PZ228509
16	*S. chinensis*	Baiyang Cha, Zhouzhi, Shaanxi	33.99522500° N/108.41760556° E	900–1014	LHM1181		PZ195093	PZ228521
17	*S. elata*	Ani Qiao, Motuo, Linzhi, Xizang	29.33028057° N/95.17890279° E	1025	MQG61		https://doi.org/10.5281/zenodo.21932899	PZ228520
18	*S. fengjiensis* sp. nov.	Lianhua Village, Fengjie, Chongqing	30.91767418° N/109.54895994° E	180.74	LHM1594_2	https://doi.org/10.5281/zenodo.19202452	PZ195095	PZ228512
19	*S. fengjiensis* sp. nov.	Lianhua Village, Fengjie, Chongqing	30.91767418° N/109.54895994° E	180.74	LHM1594_1	https://doi.org/10.5281/zenodo.19202452	https://doi.org/10.5281/zenodo.19202452	https://doi.org/10.5281/zenodo.19202452
20	*S. fengjiensis* sp. nov.	Lianhua Village, Fengjie, Chongqing	30.91767418° N/109.54895994° E	180.74	LHM1594_4	https://doi.org/10.5281/zenodo.19202452	https://doi.org/10.5281/zenodo.19202452	https://doi.org/10.5281/zenodo.19202452
21	*S. fengjiensis* sp. nov.	Lianhua Village, Fengjie, Chongqing	30.91767418° N/109.54895994° E	180.74	LHM1594_9	https://doi.org/10.5281/zenodo.19202452	https://doi.org/10.5281/zenodo.19202452	https://doi.org/10.5281/zenodo.19202452
22	*S. flavovirens*	Mingtang Shan, Yuexi, Anqing, Anhui	30.8750667° N/116.0817113° E	1046.32	LHM1307	OP703176.1	PZ195098	PZ228517
23	*S. giraldii*	Guanyin Shan, Zhouzhi, Shaanxi	33.9827778° N/108.4328862° E	1912	LHM1180		PZ195085	PZ228518
24	*S. hacquetioides*	Yajiageng Shan, Kangding, Sichuan	29.92547261° N/101.98205156° E	3584.83	LHM1166		PZ195086	PZ228523
25	*S. lamelligera*	Ximaping, Xuanen, Hubei	29.9362223° N/109.5968334° E	682	LHM1098		PZ185128	https://doi.org/10.5281/zenodo.19202452
26	*S. lamelligera*	West of Tianmu, Lin'an, Zhejiang	30.32871113° N/119.4391612° E	472	LHM1115	OP703174.1	PZ195094	PZ228513
27	*S. lamelligera*	Yesanxia, Jianshi, Hubei	30.39263889° N/109.99547233° E	1132	LHM1081		PZ179864	
28	*S. orthacantha*	Huanglong Lu, Lushan, Jiangxi	29.54758334° N/115.9653889° E	909	LHM1109	NC_072642.1	PZ195096	PZ228522
29	*S. potaninii*	Taibai Shan Shizhen, Meixian, Shaanxi	34.0103751° N/107.8235695° E	2484	LHM1200		PZ195087	PZ228519
30	*S. rubriflora*	Xiaobeigou, Jiangyuan, Baishan, Jilin	42.04085554° N/126.704289° E	662.8	LHM1310		PZ195097	PZ228514
31	*S. serrata*	Cangping Village, Huoshan, Anhui	31.17497223° N/116.18508334° E	796	LHM1110	OP703175.1	PZ195088	PZ228516

Total genomic DNA was extracted from silica‐dried leaf tissues using a modified CTAB method (Doyle and Doyle 1987). DNA integrity and purity were evaluated using 1% (w/v) agarose gel electrophoresis and a NanoDrop 2000 spectrophotometer (Thermo Scientific, Waltham, MA, USA), respectively, and DNA concentration was quantified using a Qubit 2.0 fluorometer.

For plastome sequencing, high‐quality DNA libraries were constructed and sequenced by Wuhan Benagen Tech Solutions Company Limited (Wuhan, China). Genome‐skimming sequencing was performed on the NovaSeq PE150 platform, generating 150‐bp paired‐end reads with an insert size of approximately 350 bp. The resulting sequencing reads were used for plastome assembly.

The nuclear ribosomal ITS and ETS regions were amplified separately by polymerase chain reaction (PCR). ITS was amplified using ITS5 (5′‐GGAAGTAAAAGTCGTAACAAGG‐3′) and ITS4 (5′‐TCCTCCGCTTATTGATATGC‐3′) (White et al. 1990), whereas ETS was amplified using 18S‐ETS (5′‐ACTTACACATGCATGGCTTAATCT‐3′) and Umb‐ETS (5′‐GCGCATGAGTGGTGAWTKGTA‐3′) (Baldwin and Markos 1998; Logacheva et al. 2010). The PCR products were purified and bidirectionally sequenced using the Sanger method by Sangon Biotech (Shanghai) Co. Ltd. Forward and reverse reads were assembled and manually checked using Geneious Prime v.2020.0.5. In total, we newly generated 21 ITS and 20 ETS sequences for *Sanicula*, including four ITS and four ETS sequences from the four individuals of *S. fengjiensis*. These sequences were deposited in Zenodo and their accession numbers are provided in Table 1.

For phylogenetic analyses, the four newly generated plastomes of *S. fengjiensis* were combined with 15 complete plastomes retrieved from NCBI GenBank, including 16 plastomes of *Sanicula* and three plastomes of *Eryngium* (Table 1).

#### 2.2.2 Complete Chloroplast Genomes Assembly and Annotation

Clean sequencing reads were assembled de novo using the GetOrganelle pipeline (Bankevich et al. 2012; Langmead and Salzberg 2012; Jin et al. 2020). Bandage v.5.6.0 (Wick et al. 2015) was used to visualize and manually inspect the assembly results. Chloroplast genome annotation was performed using PGA (Qu et al. 2019). Start and stop codons, as well as intron–exon boundaries, were manually corrected in Geneious Prime 2020.0.5 (Kearse et al. 2012). Circular genome maps were generated using OrganellarGenomeDRAW v.1.3.1 (Greiner et al. 2019). The annotated chloroplast genomes were deposited in Zenodo.

#### 2.2.3 Phylogenetic Analysis

To reconstruct the phylogenetic relationships within the genus *Sanicula*, 19 plastomes, 22 ITS sequences, and 21 ETS sequences, including the outgroup, were analyzed. Three *Eryngium* species were selected as outgroups for the plastome dataset, and one *Eryngium* species was selected as the outgroup for the ITS and ETS datasets, based on the phylogenetic framework proposed in a previous study (Calviño and Downie 2007). The plastome, ITS, and ETS datasets were aligned separately using MAFFT v7.221 (Katoh and Standley 2013).

Maximum‐likelihood analyses of the plastome, ITS, and ETS datasets were conducted separately using IQ‐TREE (Nguyen et al. 2015; Stamatakis and Kozlov 2020) with automatic substitution model selection and 1000 bootstrap replicates to evaluate nodal support. The analyses were run on the CIPRES Science Gateway platform (Miller et al. 2010). BI analyses were conducted in MrBayes on XSEDE v3.2.7a (Huelsenbeck and Ronquist 2001), with models selected under the Bayesian model choice setting implemented in MrBayes (nst = 6, rates = gamma).

Furthermore, the ITS and ETS gene trees were summarized to infer the ASTRAL tree (Mirarab et al. 2014). The final phylogenetic trees were visualized using Interactive Tree of Life (iTOL) v6 (Letunic and Bork 2024).

## 3 Results and Discussion

### 3.1 Morphology and Ecology

*Sanicula fengjiensis* was morphologically most similar to *S. caerulescens* , particularly in having pseudo‐racemose inflorescences. It could, however, be distinguished from *S. caerulescens* by its usually unbranched or only sparsely branched inflorescences composed predominantly of terminal capitate umbellules, mostly white petals (vs. blue to purple), and fruits lacking spinous prickles. In *S. fengjiensis*, the fruit‐surface appendages were strongly reduced, with the ribs basally fused into a thin layer and the valleculae occasionally bearing sparse scales or tubercles, whereas the fruits of *S. caerulescens* were sparsely to densely covered with short, straight spinous bristles that were basally fused into a thin sheath (Figures 1 and 2A).

**FIGURE 1:**
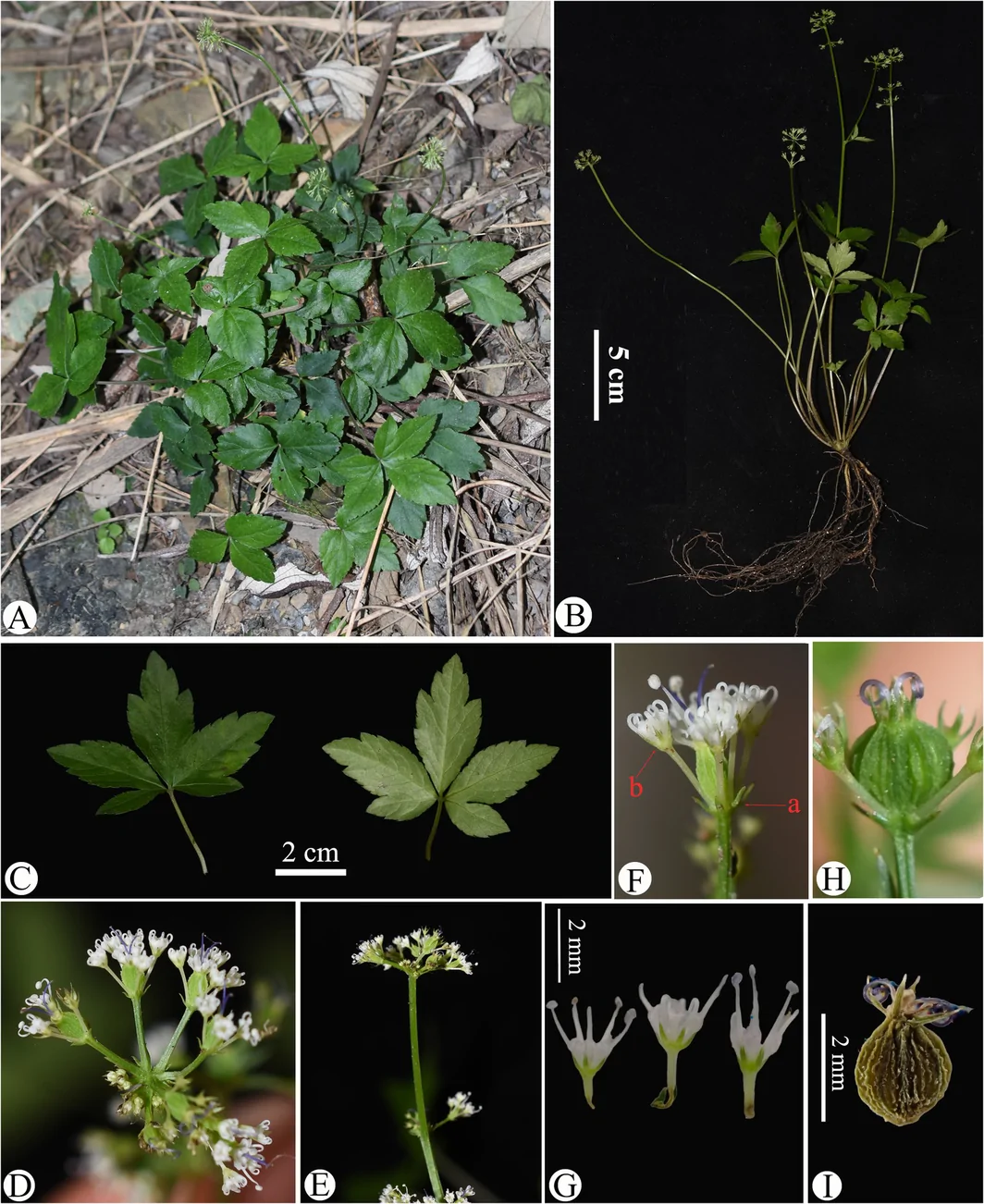
*Sanicula fengjiensis* sp. nov. in the wild (China, Chongqing, Fengjie, the type locality). (A) Habitat. (B) habit. (C) leaf (left: adaxial surface; right: abaxial surface). (D) portion of inflorescence (top view). (E) portion of inflorescence (side view). (F) umbellule (side view; a. involucellate bracteoles, b. calyx teeth). (G) staminate flowers (side view). (H) fertile flower with fruit, male pedicel, styles and calyx teeth. (I) mericarps with styles and calyx teeth. Photographed by Huimin Li.

**FIGURE 2:**
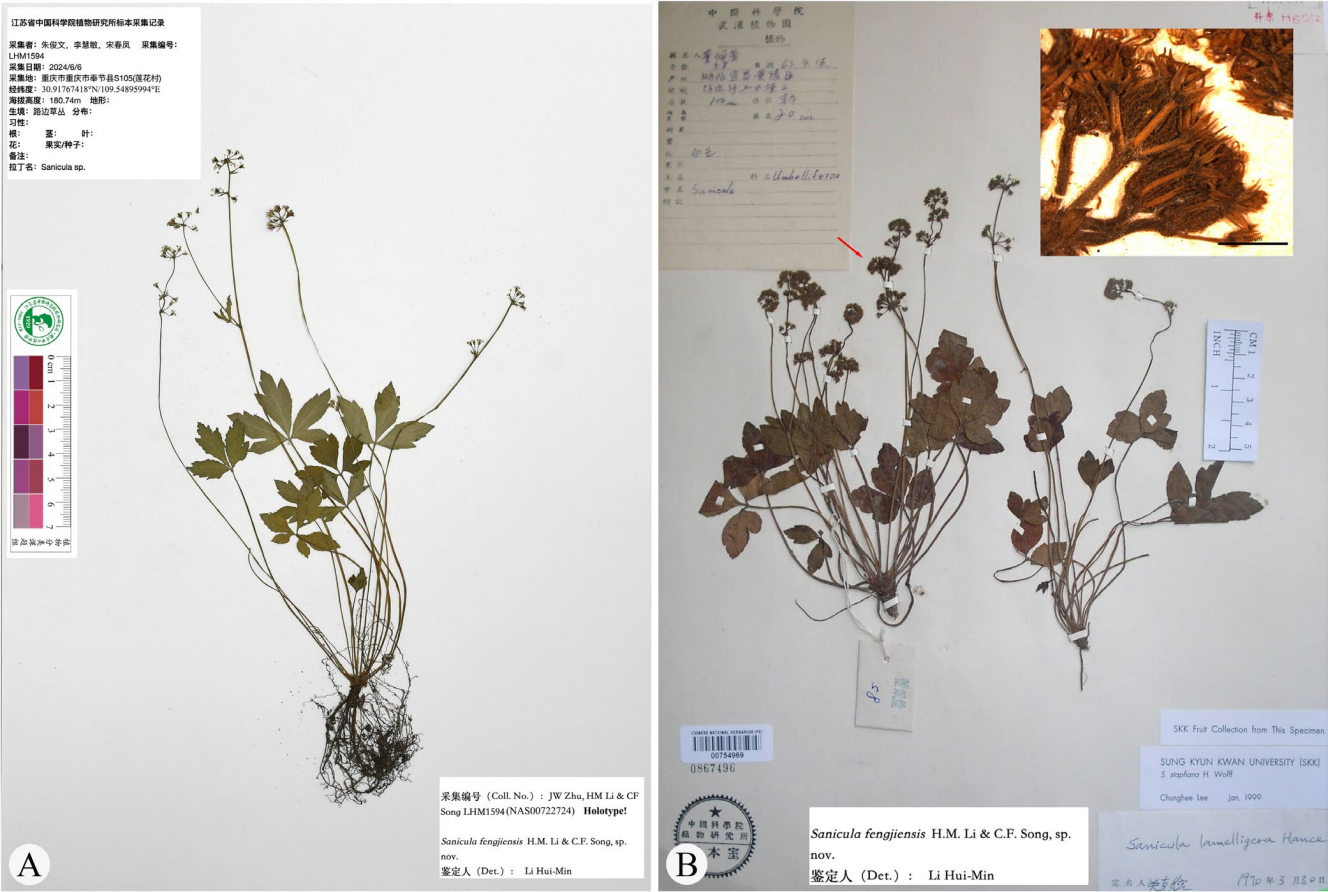
The holotype (A) and specimen (B) of *Sanicula fengjiensis* sp. nov.

Given that the nrDNA ASTRAL tree (Figure 4B) placed *S. fengjiensis* in a clade with *S. lamelligera* and *S. orthacantha*, these two phylogenetically proximate species were additionally included in the morphological comparison. Both species could also be readily distinguished from *S. fengjiensis* by their markedly different inflorescence branching patterns, together with differences in other floral and fruit characters summarized in Table 2. Quantitative analyses of 10 morphological characters across the four species further supported the distinctiveness of *S. fengjiensis*. The first two principal components accounted for 49.8% of the total morphological variation (PC1 = 28.2%; PC2 = 21.6%) (Figure 5A,B; Tables S1 and S2). Among the measured characters, maximum fruit‐surface appendage height and terminal capitate umbel diameter showed the strongest diagnostic differentiation of *S. fengjiensis* from the other three species.

**TABLE 2:** Morphological comparison of *Sanicula fengjiensis* with *S. caerulescens* , *S. lamelligera*, and *S. orthacantha*.

Characters	*S. fengjiensis*	*S. caerulescens*	*S. lamelligera*	*S. orthacantha*
Inflorescence	Pseudo‐racemose, rarely branched; capitate umbellules terminal, with the lower ones fascicled, sessile or short‐pedunculate	Pseudo‐racemose, sometimes the lower umbels in fascicles, sessile or short‐pedunculate	With 1 or 2 to several branches, sometimes subcorymbose	2–3 branched; umbels 3–8, sometimes with a shortened branch between forks or on lateral branches
Involucrate bracts	Lanceolate to ovate, acuminate, 2–3 mm long	Lanceolate to ovate, acuminate, ca. 1.6 mm long	Trisect or linear‐lanceolate, 3–6, ca. 2 mm long	Linear‐lanceolate or trisect, 2–6, 2–4.6 mm long
Rays of umbels	Terminal umbels pedunculate, 2‐ to 10‐radiate, 4.8–6.0 mm long	Terminal umbels pedunculate, 2‐ to 12‐radiate, 2.5–7.0 mm long	Terminal umbels pedunculate, 1–7‐radiate, ca. 3.5 mm long	Terminal umbels pedunculate, 1–9‐radiate, 1.8–7.5 mm long
Involucellate bracteoles	2–6, linear to lanceolate, 0.7–1.1 mm long	4–6(−8), linear to lanceolate, ca. 1 mm long	4–6, linear to lanceolate, ca. 0.6 mm long	4–6, linear to lanceolate, 0.4–1 mm long
Umbellules	5–7‐flowered	5–7‐flowered	4–7‐flowered	4–10‐flowered
Staminate flower	4–6 per umbellule; pedicels ca. 2 mm long; petals mainly white	4–6 per umbellule; pedicels 2.1–4.0 mm long; petals mainly blue to purple	3–6 per umbellule, pedicels ca. 1.5 mm long, petals white, pinkish or bluish purple	3–9 per umbellule, pedicels 0.8–3.9 mm long, petals white, pale blue or purplish red
Fertile flower	1 per umbellule, sessile	1 per umbellule, sessile	1 per umbellule, sessile	1 per umbellule, sessile
Mericarps	Globose to ellipsoid, ca. 1.8 mm long, 1.0–1.8 mm broad, prickles absent; ribs basally fused into a thin layer; valleculae occasionally bearing sparse scales or tubercles	Globose to ellipsoid, 2.0–3.0 mm long, 1.0–1.5 mm broad, sparsely to densely covered with short, straight spinous bristles; bristles usually fused at the base, forming a thin sheath	Oblong‐ovoid to ovoid, ca. 2.5 mm long, ca. 2 mm broad, variably spiculate‐lamellate, comblike	Oblong‐ovoid to ovoid, 0.7–3.5 mm long, 0.5–2.8 mm broad, ribs spinulose, furrows smooth, more or less spinulose

Maximum fruit‐surface appendage height (T10) showed particularly pronounced differentiation among species (Kruskal–Wallis test, BH‐adjusted *p* < 0.001, *ε* ^2^ = 0.525; Figure 5C). In *S. fengjiensis*, this character ranged from 0.033 to 0.101 mm, with no overlap with the observed ranges of *S. caerulescens* (0.253–0.625 mm), *S. lamelligera* (0.152–0.447 mm), or *S. orthacantha* (0.265–0.800 mm) (Figure 5F and Table S2). Terminal capitate umbel diameter (T3) also differed significantly among species (one‐way ANOVA, BH‐adjusted *p* < 0.001, *η* ^2^ = 0.378; Figure 5D). Although the overall observed ranges showed some overlap, the interquartile range of *S. fengjiensis* was completely separated from those of the other three species (Figure 5F). The number of primary inflorescence branches (T2) provided additional differentiation from *S. lamelligera* and *S. orthacantha*, although it did not significantly distinguish *S. fengjiensis* from *S. caerulescens* (Figure 5E). In contrast, several other quantitative characters showed considerable interspecific overlap and therefore had lower diagnostic value when considered individually.

Taken together, the quantitative differences and qualitative characters of the inflorescences, petals, calyx teeth, and fruit surface distinguished *S. fengjiensis* from both the morphologically similar *S. caerulescens* and the phylogenetically proximate *S. lamelligera* and *S. orthacantha*. Detailed morphological comparisons among the four species were provided in Table 2.

According to available distributional records, the four species also differed geographically and ecologically. *S. caerulescens* was widely distributed in China (Chongqing, Guangxi, Hubei, Hunan, Sichuan, and Yunnan) and Vietnam (Hà Giang), occurring mainly at elevations of 400–1700 m (Li et al. 2024). *S. lamelligera* was widely distributed in central, eastern, southern, and southwestern China and southern Japan, where it occurred in forests on mountain slopes and along ravines and streams at elevations of 450–1600 m (Li and Song 2022). *S. orthacantha* was widely distributed in China (Anhui, Chongqing, Fujian, Gansu, Guangdong, Guangxi, Guizhou, Hubei, Hunan, Jiangxi, Shaanxi, Sichuan, Taiwan, Yunnan, and Zhejiang) and Vietnam, occurring mainly on forested mountain slopes and along ravines and streams at elevations of 600–2140 m (Li et al. 2022). At the time of this study, *S. fengjiensis* was documented only from Chongqing and Hubei (Figure 6), with known extant populations occurring at relatively low elevations of approximately 100–200 m on mountain slopes.

### 3.2 Characteristics of Plastomes and Phylogenetic Analysis

The complete plastomes of four *Sanicula fengjiensis* accessions were all 155,501 bp in length (https://doi.org/10.5281/zenodo.19202452; A representative plastome map is shown in Figure 3). All four plastomes exhibited the typical quadripartite structure, comprising a large single‐copy (LSC) region, a small single‐copy (SSC) region, and two inverted repeat regions (IRa and IRb) (representative plastome characteristics are shown in Table 3).

**FIGURE 3:**
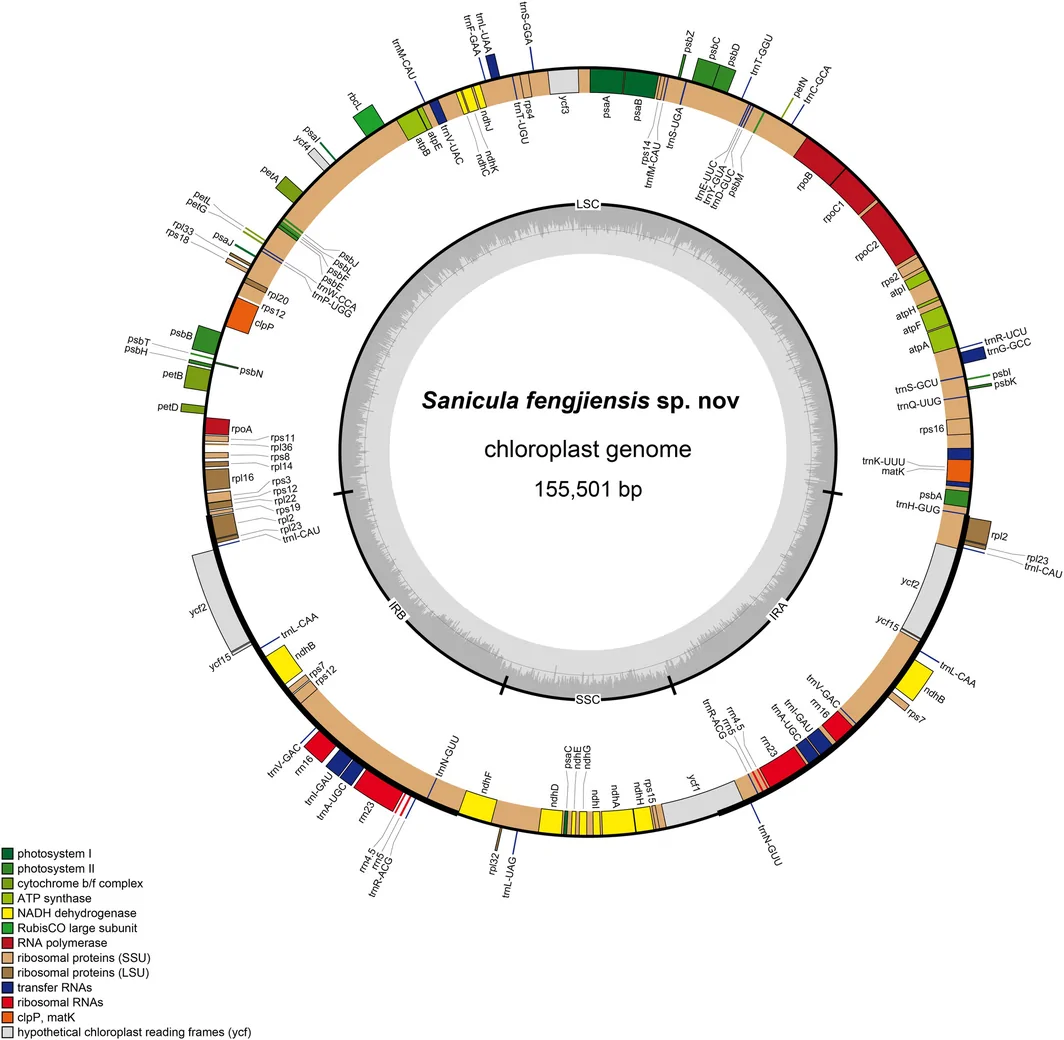
Circular gene map of a representative complete plastome of *Sanicula fengjiensis* sp. nov. (LHM1594‐2). Genes shown outside the circle are transcribed clockwise, whereas those inside are transcribed counterclockwise. Genes are color‐coded according to different functional groups. The dark gray and light gray plots in the inner circle indicate GC content and AT content, respectively. IR, inverted repeat; LSC, large single‐copy region; SSC, small single‐copy region.

**TABLE 3:** Plastome characteristics of a representative accession of *Sanicula fengjiensis* sp. nov.

Characteristic	*Sanicula fengjiensis* sp. nov.
Total length (bp)	155,501
GC%	38.2
LSC length (bp)	85,923
SSC length (bp)	17,098
IR length (each; bp)	26,240
Total genes	132
Protein‐coding genes	87
rRNA genes	8
tRNA genes	37

*Note:* The four sampled accessions had identical values for the plastome characteristics listed; therefore, a single representative set of values is presented.

Phylogenetic analyses based on plastome and combined ITS and ETS datasets consistently recovered the sampled accessions of *S. fengjiensis* as a distinct clade, although its relationships with other *Sanicula* species differed between the two datasets. In the plastome phylogeny, the four sampled accessions of *S. fengjiensis* formed a strongly supported clade (BS = 100, PP = 1.00; Figure 4A). This clade was resolved as sister to a larger lineage containing *S. rugulosa* Diels, *S. brevispina* (H.Boissieu) B.N. Song & X.J. He, *S. orthacantha*, *S. lamelligera*, and *S. caerulescens* , although support for this relationship was relatively low (BS = 62, PP = 0.60).

**FIGURE 4:**
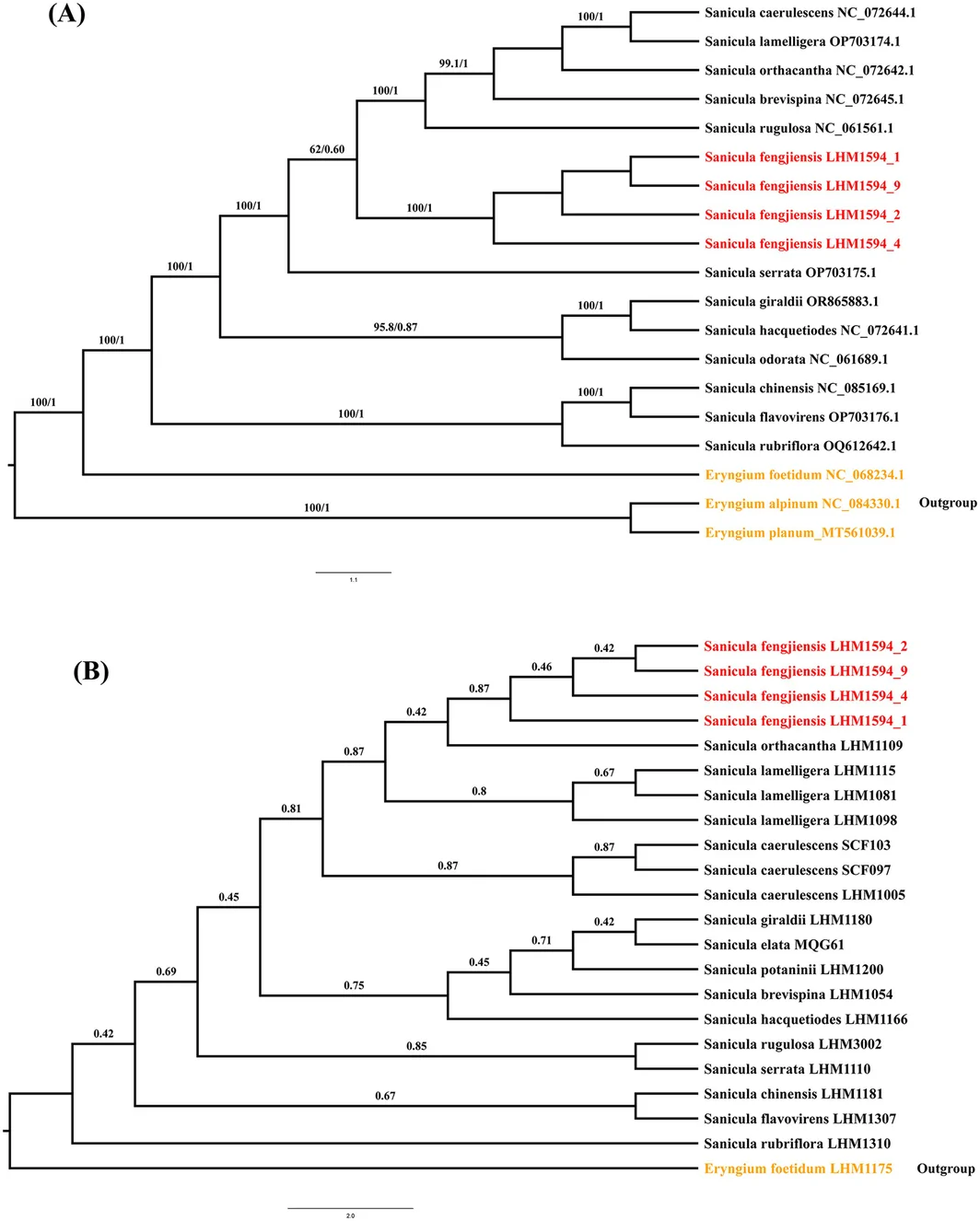
Phylogenetic trees inferred from both complete chloroplast genomes and nrDNA markers are shown. (A) Tree based on complete chloroplast genomes of 16 *Sanicula* samples and three *Eryngium* species, inferred using Maximum Likelihood (ML) and Bayesian Inference (BI); bootstrap support and posterior probability are shown on branches as ML/BI. (B) ASTRAL tree based on ITS and ETS sequences; local posterior probabilities (LPP) are shown on the branches.

In the ASTRAL tree inferred from ITS and ETS gene trees, the four accessions of *S. fengjiensis* also formed a clade (LPP = 0.87; Figure 4B). This clade was placed with *S. orthacantha*, although their immediate relationship received low support (LPP = 0.42), and the resulting lineage was grouped with *S. lamelligera* with higher support (LPP = 0.87). This broader lineage was sister to the clade comprising the three sampled populations of *S. caerulescens* (LPP = 0.81).

The plastome and nrDNA datasets therefore recovered different placements of *S. fengjiensis*, indicating topological discordance between the plastome and nrDNA phylogenies. The underlying cause of this discordance cannot be determined from the present data alone. Nevertheless, the sampled accessions of *S. fengjiensis* consistently formed a coherent clade in both analyses. Together with the diagnostic morphological differences from *S. caerulescens* , *S. lamelligera*, and *S. orthacantha*, these results supported the recognition of *S. fengjiensis* as a distinct species.

## 4 Taxonomic Treatment

*Sanicula fengjiensis* H.M. Li & C.F. Song, sp. nov. (Figures 1 and 2).

### 4.1 Type

CHINA. Chongqing municipality: Fengjie County, Lianhua village, along roadsides, in forest understories, and on stony slopes, 30.91767418° N, 109.54895994° E, 180.74 m alt., 6 June 2024, LHM1594 (fl., holotype NAS00722724!, isotype NAS00722725!).

### 4.2 Diagnosis

*Sanicula fengjiensis* is morphologically similar to *S. caerulescens* in having pseudo‐racemose inflorescences, but differs in having usually unbranched or sparsely branched inflorescences, predominantly white petals (vs. blue to purple), and fruits lacking spinous prickles, with the ribs basally fused into a thin layer and the valleculae occasionally bearing sparse scales or tubercles (vs. fruits sparsely to densely covered with short, straight spinous bristles that are basally fused into a thin sheath). It is also readily distinguished from the phylogenetically proximate *S. lamelligera* and *S. orthacantha* by its much less extensively branched inflorescences and distinctive fruit‐surface ornamentation (Table 2).

### 4.3 Description

Perennial. Rhizome, oblique rootstock, bearing fibrous roots. Stems 1–8, slender and erect, 18–28 cm tall. Basal leaves 7–24, long petiolate; petioles 1.5–13 cm long; blade glabrous adaxially and abaxially, 1–7 cm long, 1–7.5 cm wide, cordate suborbicular, ternate, segments distinctly petiolate; median segments obovate to ovate‐lanceolate, distinctly shallowly trilobed, petiolate; lateral segments obliquely ovate, bilobed to the middle or only notched or rarely entire; margins irregularly crenulate serrate with spinose teeth. Cauline leaves absent or nearly absent; degenerating into sheathing, trisect or undivided, bract‐like, ca. 2.0 mm long, subsessile or sessile. Inflorescence rarely branched, pseudo‐racemose, consisting of capitate umbellules only, terminal; lower umbels fascicled, sessile or short‐pedunculate; involucrate bracts lanceolate to ovate, acuminate, 2–3 mm long; terminal umbels pedunculate, with 2–10 rays, rays 4.8–6.0 mm long; involucellate bracteoles 2–6, linear to lanceolate, 0.7–1.1 mm long. Staminate flowers 4–6 per umbellule; pedicels ca. 2 mm long; petals mainly white. Fertile flowers 1 per umbellule, sessile; calyx teeth 4–8, linear to lanceolate, acute, 0.6–1.2 mm long; styles 2.0–3.6 mm long, recurved. Mericarps globose to ellipsoid, ca. 1.8 mm long, 1.0–1.8 mm broad, along ribs fused at base into a thin layer; valleculae occasionally bearing sparse scales or tubercles; prickles absent; mericarps dorsally flattened, orbicular in cross‐section.

### 4.4 Phenology

Flowering from late March to early May. Fruiting from late March to early June.

### 4.5 Etymology

The epithet “fengjiensis” is derived from the type locality, Fengjie County, Chongqing Municipality, China. The Chinese name given is Feng Jie Bian Dou Cai (奉节变豆菜).

### 4.6 Distribution and Habitat

*Sanicula fengjiensis* is currently known from its extant population at the type locality, that is, Lianhua Village in Fengjie County, Chongqing Municipality, China, and from a historical herbarium record collected from Yichang City, Hubei Province, China (Figure 5). It grows along roadsides, in forest understories, and on stony slopes at altitudes of 100–200 m.

**FIGURE 5:**
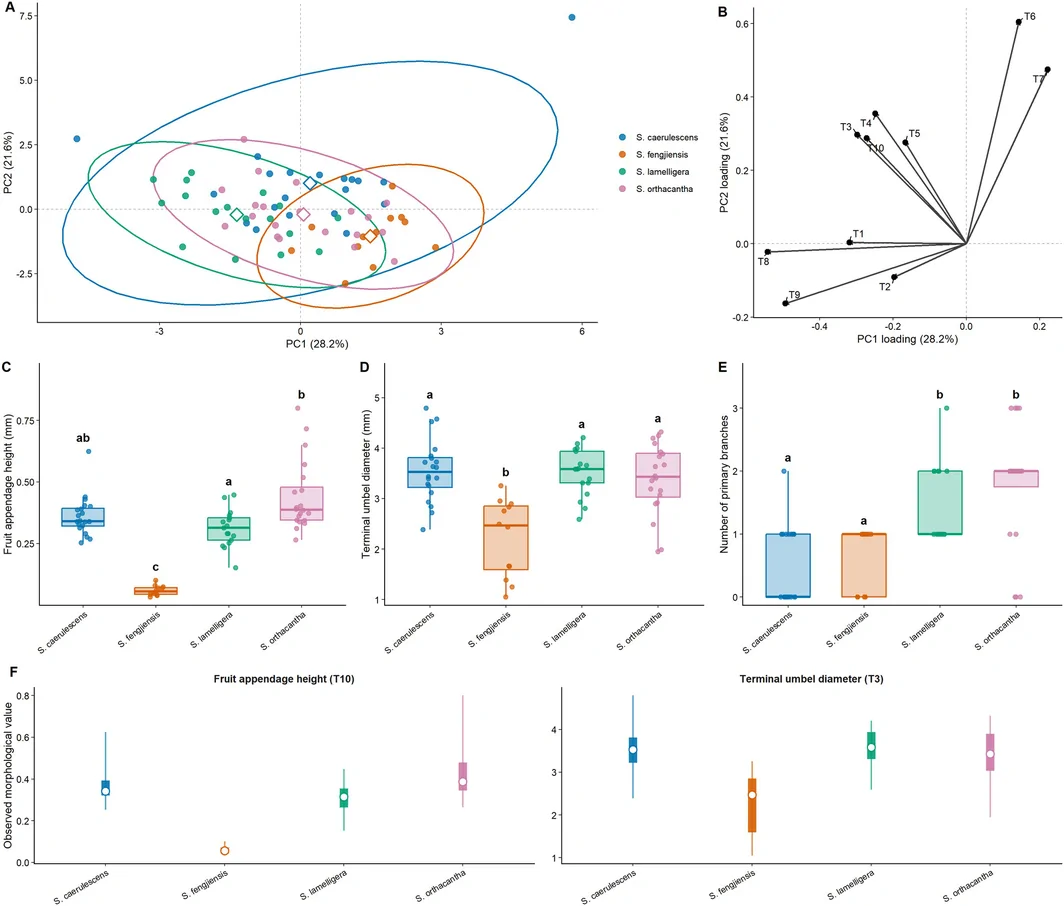
Quantitative morphological differentiation among *Sanicula fengjiensis*, *S. caerulescens* , *S. lamelligera*, and *S. orthacantha*. (A) PCA based on ten quantitative morphological characters. (B) Loadings of T1–T10 on PC1 and PC2. (C–E) Variation in fruit‐surface appendage height (T10), terminal capitate umbel diameter (T3), and number of primary inflorescence branches (T2), respectively; different lowercase letters indicate significant pairwise differences (*p* < 0.05). (F) Observed ranges of T10 and T3; thin lines indicate min–max, thick bars indicate Q1–Q3, and open circles indicate medians.

**FIGURE 6:**
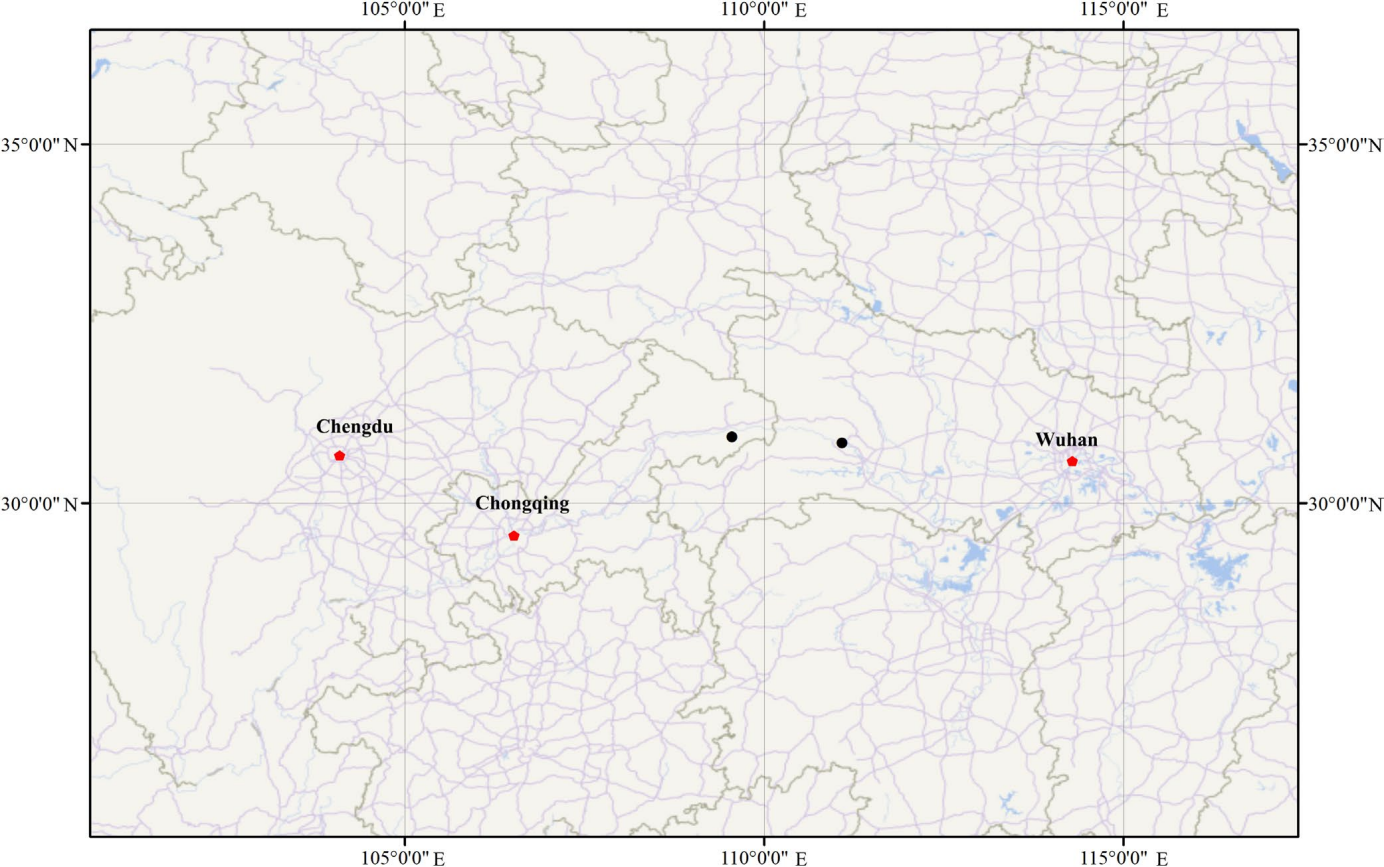
Distribution map of *Sanicula fengjiensis* sp. nov. (●).

### 4.7 Preliminary Conservation Status

Based on extensive examinations of herbarium specimens and our field surveys, *Sanicula fengjiensis* is rare in its type locality and adjacent areas. To date, only one extant population and one historical locality are known: one observed in the wild and one identified from specimen records. As its full distribution range, population dynamics, and potential threats remain unclear, we tentatively assess this species as Data Deficient (DD) according to the IUCN Red List Categories and Criteria (IUCN Standards and Petitions Committee 2024).

## Author Contributions

**Hui‐Min Li:** data curation (equal), formal analysis (equal), investigation (equal), software (equal), visualization (equal), writing – original draft (equal). **Ji‐Min Liao:** formal analysis (equal), methodology (equal), writing – original draft (equal). **Xu‐Dong Ma:** formal analysis (equal), methodology (equal). **Chun‐Feng Song:** conceptualization (equal), formal analysis (equal), investigation (equal), methodology (equal), project administration (equal), supervision (equal), writing – original draft (equal), writing – review and editing (equal).

## Funding

This work was financially supported by the National Natural Science Foundation of China (Grant 32370220) and the Natural Science Foundation of Jiangsu Province (Grant BK20200294).

## Conflicts of Interest

The authors declare no conflicts of interest.

## Supporting information


**Table S1:** Individual‐level measurements of 10 quantitative morphological characters in four *Sanicula* species.

**Table S2:** Summary statistics of 10 quantitative morphological characters in four *Sanicula* species.

## Data Availability

The plastome sequences generated in this study have been deposited in Zenodo and are publicly available at https://doi.org/10.5281/zenodo.19202452. Newly generated nuclear ribosomal DNA sequences (ITS and ETS) have been deposited in either GenBank or Zenodo, and their accession numbers are provided in Tables 1 and S1. Detailed collection information and sequence identifiers are also provided in these tables.
